# Direct Synthesis of HKUST-1 onto Cotton Fabrics and Properties

**DOI:** 10.3390/polym14204256

**Published:** 2022-10-11

**Authors:** Braian Lobo da Costa, Isadora Letícia Aparecida Ataide Rosa, Vitória Hipolito Silva, Qiuyue Wu, Rafael Block Samulewski, Fabio Alexandre Pereria Scacchetti, Murilo Pereira Moisés, Manuel J. Lis, Fabricio Maestá Bezerra

**Affiliations:** 1 Textile Engineering Coordination (COENT), Universidade Tecnológica Federal do Paraná (UTFPR), Campus Apucarana, 635 Marcilio Dias St., Apucarana 86812-60, Brazil; 2 Chemistry Coordination (COLIQ), Universidade Tecnológica Federal do Paraná (UTFPR), Campus Apucarana, 635 Marcilio Dias St., Apucarana 86812-60, Brazil; 3 Institute of Textile Research and Cooperation of Terrassa, Polytechnic University of Catalonia, C/Colom 15, 08222 Terrassa, Barcelona, Spain

**Keywords:** cotton, metal-organic framework, surface modification, antimicrobial, textile finish

## Abstract

Metal-organic frameworks are crystalline nanostructures formed by a metal interspersed by an organic binder. These metal-organic materials are examples of nanomaterials applied to textile material in search of new functionalized textiles. Cotton is a cellulosic fiber of great commercial importance, and has good absorption capacity and breathability; however, due to these characteristics, it is susceptible to the development of microorganisms on its surface. This work aims to analyze how the direct synthesis of HKUST-1 in cotton fabric modifies the chemical and physical properties. The material obtained was characterized by scanning electron microscopy to obtain its morphology, by spectrophotometry CIE L*a*b* to verify the color change, by a biological test to verify its resistance to microorganisms and, finally, by a unidirectional traction test to verify the change in its mechanical resistance. Thereby, it was possible to observe the formation of MOFs with the morphology of nanorods, and also, with regard to HKUST-1 in the cotton fabric, when applied, an elimination percentage higher than 99% was observed for both bacteria, *E. coli* and *S. aureus*. The presence of MOF was detected even after washing, however, the loss of 75% in the mechanical resistance of the material makes its potential for textile finishing unworkable.

## 1. Introduction

The research and development of a new generation of antimicrobial materials to mitigate the spread of antibiotic resistance has become imperative, as pathogens have created a resistance to most antimicrobials available on the market [1,2,3]. Metallic nanomaterials have stood out as bactericidal agents due to the multiple mechanisms of action, which make it difficult for microorganisms to mutate [4,5,6,7,8]. In the context of the various possibilities of the application of these materials, the textile industry has stood out for its intense consumption of these bactericidal agents [9,10,11,12,13].

The finishing process in the textile industry aims to improve or add properties to the material subjected to treatment, through physical, chemical, or biological methods [14]. According to Yetisen and coworkers, nanoengineering appears as a new partner in textile finishing, because, from it, it is possible to obtain functionalized textiles without change in comfort characteristics [15].

An example of functionalization is the use of metal-organic networks (Metal Organic Framework—MOF). These materials have a metallic core interspersed with an organic ligand, creating a tridimensional crystalline structure [16]. In addition to the extensive surface area and storage capacity, which give them the ability to be used in drug delivery or gas storage, MOFs can have bactericidal characteristics, depending on the metal chosen for synthesis, making them excellent candidates for the antimicrobial finish [17]. However, this material has a low fixing capacity to other materials, making the choice of fiber where the finishing will be carried out, extremely important [18].

Cotton is the second most consumed textile fiber in the world, behind only polyester [19,20]. Its good breathability and absorption capacity make it a promising site for the proliferation of microorganisms [21]. The growth of these beings can cause a degradation of the substrate, and even cause possible contamination [22,23]. Therefore, this work seeks the direct synthesis of HKUST-1 in cotton fabrics, analyzing how it influences the properties of the textile material.

## 2. Materials and Methods

All reagents used were of analytical grade: copper (II) nitrate (Vetec, 98%, Rio de Janeiro, Brazil), trimesic acid (Vetec, 98%, Rio de Janeiro, Brazil), and non-ionic detergent (Golden Technology, São José dos Campus, Brazil). The fabric was 100% cotton with 210 ± 5 g m^−2^ weight.

Samples were named based on their synthesis of time and temperature, with the first two digits denoting the time and the other digits the temperature. The unfinished sample was named Blank.

### 2.1. In Situ Synthesis of HKUST-1

Before functionalization, samples were cut to size 6 × 20 cm and rinsed with a bath ratio of 1:40 (m:v), and 1 mL L^−1^ of a non-ionic detergent, for 10 min at 40 ± 2 °C.

The synthesis of MOF was based on the hydrothermal processes described by Wang et al. [22] and Lis et al. [24]. A sample was placed in an 18.67 g L^−1^ copper nitrate solution and stirred for 30 min at 40 ± 2 °C, then 15 mL of a 28.00 g L^−1^ ethanolic solution of trimesic acid was added. The resulting solutions were kept at 80 ± 2 °C and 100 ± 2 °C for 18, 24, and 48 h, totaling 6 experiments, being named by the following codes: 1880, 2480, 4880, 18,100, 24,100 and 48,100, where the first two numbers represent the processing time and the others the temperature.

After the reaction interval, the samples were rinsed at room temperature to remove the excess reagents and then dried in an oven at 60 ± 2 °C for 24 h. Moreover, the residual baths and the post-reaction rinse baths for each time were filtered, to obtain the residual MOF present in the bath. To verify the washing resistance, a process adapted from ABNT NBR ISO 105 C06 was carried out after sample drying. [25] Samples were rinsed in a bath of 1:40 (m:v) ratio of distilled water for 5 min at room temperature and dried at room temperature.

### 2.2. Characterization

Scanning Electron Microscopy (SEM) was performed with a Quanta 250 microscope (FEI Company, Eindhoven, The Netherlands). Samples were attached to the support with carbon tape and metalized with gold. The generated images were used to verify the morphology and amount of surface MOF anchorage, using the Gimp image software, and the (longitudinal) size of the nanorods was determined.

X-ray diffractometry (XRD) analyzes were performed with the D2 Phaser Diffractometer equipment from Bruker^®^ (Allentown, PA, USA) using CuKα radiation (1.54 Å/8.047 keV), an angular range (2q) from 5 to 60 degrees, and an angular increment of 0.033 degrees s^−1^ (2 degrees min^−1^).

Delta Vista 450 G A Spectrophotometer (Sao Paulo, Brazil), 10° observer, Iluminate D_65_ and 2 mm aperture was used to analyze the color change of the sample. Each sample was measured three times, generating chromatic coordinates, arranged in the CIE Lab space. The value of L* indicates the luminosity of the samples. The a* coordinate represents the proximity to red for positive values, and green for negative values, whereas, with respect to the b* coordinate, positive values indicate a color close to yellow, while blue indicates negative values.

### 2.3. Mechanical Properties

The variation in the mechanical strength of the samples was verified by a uniaxial tensile test with a constant displacement rate of 20 mm min^−1^, in the warp direction, using the WDW-300E universal testing machine by the Time-Shijin Group (Narwood, MA, USA). Samples were placed in the grips in the shape of an hourglass, and, from the generated data, the mean and standard deviation of the applied force data were calculated.

### 2.4. Evaluation of Antibacterial Properties

#### 2.4.1. Minimum Inhibitory Concentration (MIC) and Minimum Bactericidal Concentration (MBC)

The minimum inhibitory concentration (MIC) and minimum bactericidal concentration (MBC) of HKUST-1 crystals were determined for the bacteria *Escherichia coli* (*E. coli*) ATCC^®^ 35218TM and *Staphylococcus aureus* (*S. aureus*) ATCC^®^ 25923TM in accordance with what is described in the standard Clinical and Laboratory Standards Institute (CLSI) for bacteria.

The MIC value was established, as the concentration of bacteria did not show any growth, determined visually with Resazurin dye (7-hydroxy-3H-phenoxazin-3-one-10-oxide) broadly used as a reliable indicator of cell viability. Then, each dilution was seeded in an agar plate and incubated at 35 ± 2 °C for 24 h. The growth of microorganisms was analyzed, and the MBC value was established for the smallest dilution that did not show any growth in agar plate.

#### 2.4.2. Bacterial Reduction

The antibacterial activity of the textile sample functionalized with HKUST-1 was tested in accordance with the quantitative test method ASTM E2149-13 (adapted), using selective/differential media by agar diffusion methods, MacConkey Agar (Kasvi^®^) for *E. coli* ATCC^®^ 35218TM and Mannitol Salt Agar (Kasvi^®^) for *S. aureus* ATCC^®^ 25923TM. The growth of a fresh shake culture of bacteria in sterile Tryptic Soy Broth (Kasvi ^®^) at 35 ± 2 °C for 24 h was necessary to perform the test. The culture was diluted in sterile solution (0.3 mM KH2PO4) with a concentration of 1.5 − 3.0 × 10^5^ CFU (Colony Forming Units) mL^−1^ or 0.5 McFarland.

The samples (20 × 20 mm), sterilized by ultra-violet (UV) radiation, were placed in a flask and inoculated with the suspension of microorganisms (50 mL) at 110 RPM, for 1 h, and immediately serially diluted, with each sample plated in duplicate. As a positive control, there was a flask with 50 mL of buffer and the same CFUs mL^−1^; however, serial dilutions and the standard plate count techniques from the “inoculum only” occurred at “0” time. The results were expressed as the average of CFUs mL^−1^, according to the test method. The log bacterial reduction regarding the microbial growth was determined according to the following equation:

Log_10_ Bacterial Reduction = Log_10_ (B) − Log_10_ (A) (1)

where, A indicates the number of CFU mL^−1^ for the flask containing the functionalized sample after the contact time and B represents the number of CFUs mL^−1^ for the “inoculum only” at “0” time.

#### 2.4.3. Time-Kill Kinetics Test

Moreover, the antibacterial activity of the textile sample functionalized with HKUST-1 was tested according to the time-kill assay ASTM E2315 (adapted), used to study the activity against microorganisms and to determine the bactericidal or bacteriostatic activity over time.

The test followed the same conditions as the Bacterial log reduction test (sample sizes, concentration of microorganisms in suspension, and other details). Then, aliquots were withdrawn at time 0 (before action), and after 5, 10, 20, 30, 45, and 60 min, they were immediately serially diluted, with each sample plated in duplicate, and cultured at TSA plates further incubated at 35 ± 2 °C for 24 h. Colonies of surviving bacteria were counted and reported as a mean standard deviation (S.D.) according to standards.

## 3. Results

### 3.1. Characterization

**Figure 1:**
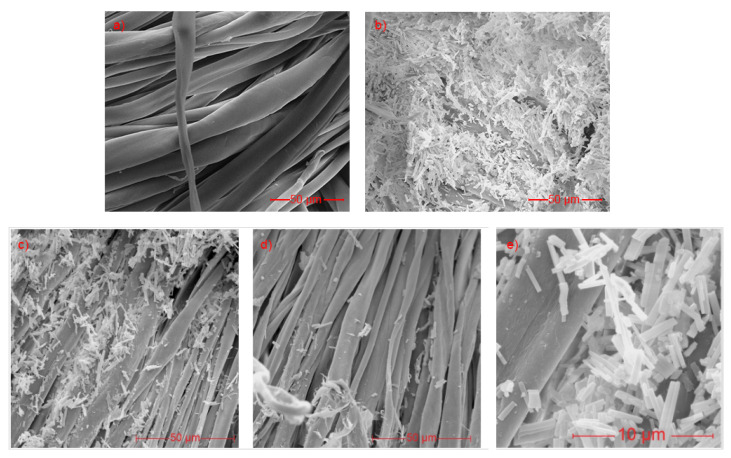
SEM images: (**a**) cotton untreated, (**b**) HKUST-1 nanorods, (**c**) cotton treated before washing and (**d**) after washing. (**e**) Highlight to HKUST-1 nanorods on the cotton.

Scanning electron microscopy (SEM) allows the observation of the textile surface. An untreated cotton must have the fibers in their natural state, with the surface being smooth and the fiber twisting along its longitudinal axis. In the SEM images of the treated cotton, it is possible to observe the HKUST-1 crystals formed on the surface with size 8.86 ± 1.34 µm. In some points of the fiber, the cotton no longer presents a smooth surface and starts to present a rougher surface. This detail is linked to the acid hydrolysis caused by the reagents used in the MOF synthesis. Figure 1 shows images of cotton untreated and treated with HKUST-1. It is possible to observe that MOF is still present on the surface of the cotton after the washing process, but a decrease in the amount of MOFs is noticeable.

At the usual synthesis temperatures of HKUST-1, the most common morphology found is octahedral, while lower temperatures lead to the shape of cubes with sharp edges. However, the morphology of HKUST-1 that was found showed the shape of a nanorod, as shown in Figure 1c. According to Yang and coworkers, variations in the concentration of organic ligands can easily change the morphology of a MOF [26]. Moreover, according to them, the morphology of nanorods presents an excellent surface area and crystallinity. It is worth mentioning that, combined with the concentrations of organic ligands at a synthesis temperature lower than the usual ones and with the longer reaction time, this may justify the increase in the crystallinity of the morphology found. Tehrani et al. [27] and Martínez et al. [28] report synthesis processes for MOF, in which the formation of HKUST-1 nanorods occurred. These same authors describe the similarity between zinc and copper MOFs, which reinforces the results found by Yang et al. [26] It is also likely that the interaction between MOF/reagents and cotton cellulose leads to an unusual shape of the MOFs.

The results of the uniaxial test show a considerable decrease in the tensile strength of the textile materials, as can be observed in Table 1. It is observed that the temperature and the reaction time negatively influence the strength of the samples, which is related to the acidic character of the synthesis reactions, in which the cotton fabric interacts with copper nitrate (pH 4) and, after the addition of trimesic acid, the pH decreases to 2. According to the literature, with regard to cotton, in the presence of acid in combination with the rise in temperature, there is a loss in its mechanical strength, due to the hydrolysis of cellulose, as the polymer chain of the fiber breaks due to the breakage of the 1,4-glycosidic bond [29,30,31]. This depolymerization ends up decreasing the number of intramolecular interactions between the cellulose polymers, resulting in a loss of strength.

From the data obtained through color measurement, the mean and standard deviation of the three variables of the L*a*b* color space were calculated. This process is necessary due to the variability that the measurements present, even within the same sample. This variability is demonstrated by the standard deviation, which is as close to zero as the smaller data variability. Through the analysis of the average, it was possible to identify the color variation of the fabric, and, in Table 2, it is possible to observe the L*a*b* values regarding all samples. From these coordinates, the color difference (DE) is calculated, and this value symbolizes the distance that the coordinate is in relation to a point in the three-dimensional space L*a*b*, thus being the distance between the coordinates of the finished samples and the unfinished white cotton fabric, as can be observed in Table 2. According to Gascon et al., HKUST-1 has a characteristic bluish color, which justifies the samples with the finish having a color variation tending to bluer tones in relation to the untreated white substrate. [32] The more yellowish tones (+b) presented by the samples synthesized at 100 °C can be justified by cellulose degradation. Samples 2480 and 4880 show the best results for the blue color coordinate (−a), and this indicates that the HKUST-1 anchorage is more efficient under these conditions.

**Figure 2:**
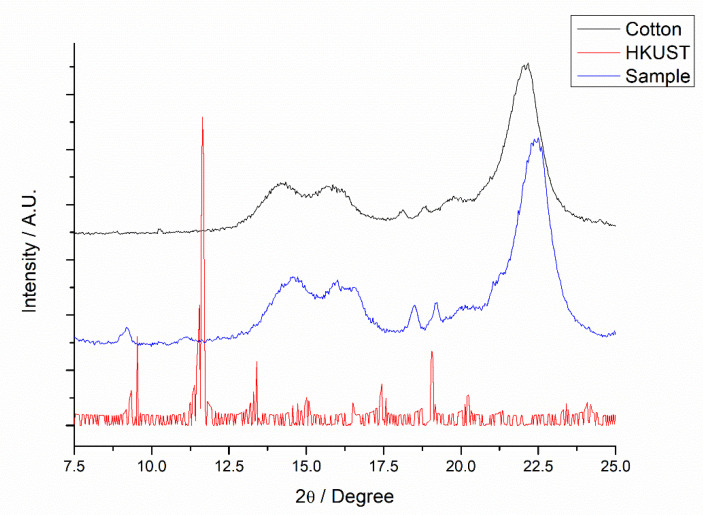
XRD patterns of fabric (-Cotton), treated fabric 2480 (-Sample) and MOF (-HKUST).

Figure 2 shows XRD patterns of cotton, sample 2480, and residual HKUST-1 collected by filtration. The diffraction peaks of the MOF are in agreement with the data presented in the literature, with characteristic diffraction peaks at 9.4°, 11.5°, 13.3° 15.0°, and 17.4° for CuKα radiation source. [33] However, due to the differences in size and concentration, since cellulose has extensive polymeric chains, it was not possible to observe the diffraction peaks of the MOF after insertion into cotton. In the fabric sample, the diffraction peaks characteristic of cotton can be observed at 2θ = 14.7°, 16.5°, and 22.4°, indicating that the main component was cellulose. [22] After MOF insertion, there is a noticeable modification in the XRD pattern profile, indicating structural changes. According to the Segual equation, which relates the diffraction peaks, 21.5 and 22.4 degrees, the crystallinity of the sample can be calculated and compared [34].

When associating the results for cotton (57.2%) and those for sample 2480 (26.9%), a decrease in crystallinity was observed. Literature data indicate that cotton crystallinity without modification is between 63 and 77%. The lower than expected value for the cotton sample was probably caused by the fact that the fabric was not washed before the analysis, and the decrease in crystallinity occurs due to the presence of waxes used in the loom process to reduce friction. In comparison with the data for sample 2480, it is possible to observe a large decrease. This occurs both because of the insertion of MOF on the surface, which grounds disorder, and as a function of the acid attack that occurs with the direct synthesis of MOF in the tissue, which corroborates the decrease in tensile strength in samples with longer time and temperature novel.

### 3.2. Evaluation of Antibacterial Properties

For all antibacterial assays, the sample (2480) was used because of its better finishing results. Table 3 presents the results regarding the minimum inhibitory concentration (MIC) and minimum bactericidal concentration (MBC) of the solutions tested for *E. coli* ATCC^®^ 35218TM and *S. aureus* ATCC^®^ 25923TM. The solutions were prepared with an initial concentration of 10 mg mL^−1^ for each substance.

The HKUST-1 dispersion obtained bacteriostatic activity against *E. coli,* since it inhibited microbial growth with a concentration of 78.125 μg mL^−1^ or higher. However, it has eliminated all the microorganisms with a concentration of 5 mg mL^−1^ or higher. On the other hand, *S. aureus* presented more resistance, HKUST-1 also obtained bacteriostatic activity, inhibited microbial growth with a concentration of 156.25 μg mL^−1^ and did not eliminate the microorganisms even at the highest initial concentration tested.

Its antimicrobial activity can result from the guests incorporated in the particle voids or from the properties of the metal component in the framework. Possibly, the degradation of the MOF releases the components in the form of metal or cations in aqueous solution. [35]

Table 4 shows the results of the antimicrobial activity (quantitative method) for *E. coli* ATCC^®^ 35218TM and *S. aureus* ATCC^®^ 25923TM for textile samples functionalized with HKUST-1. The samples presented an almost complete reduction of microorganisms compared with the positive control, which demonstrated an expected growth of microorganisms.

**Figure 3:**
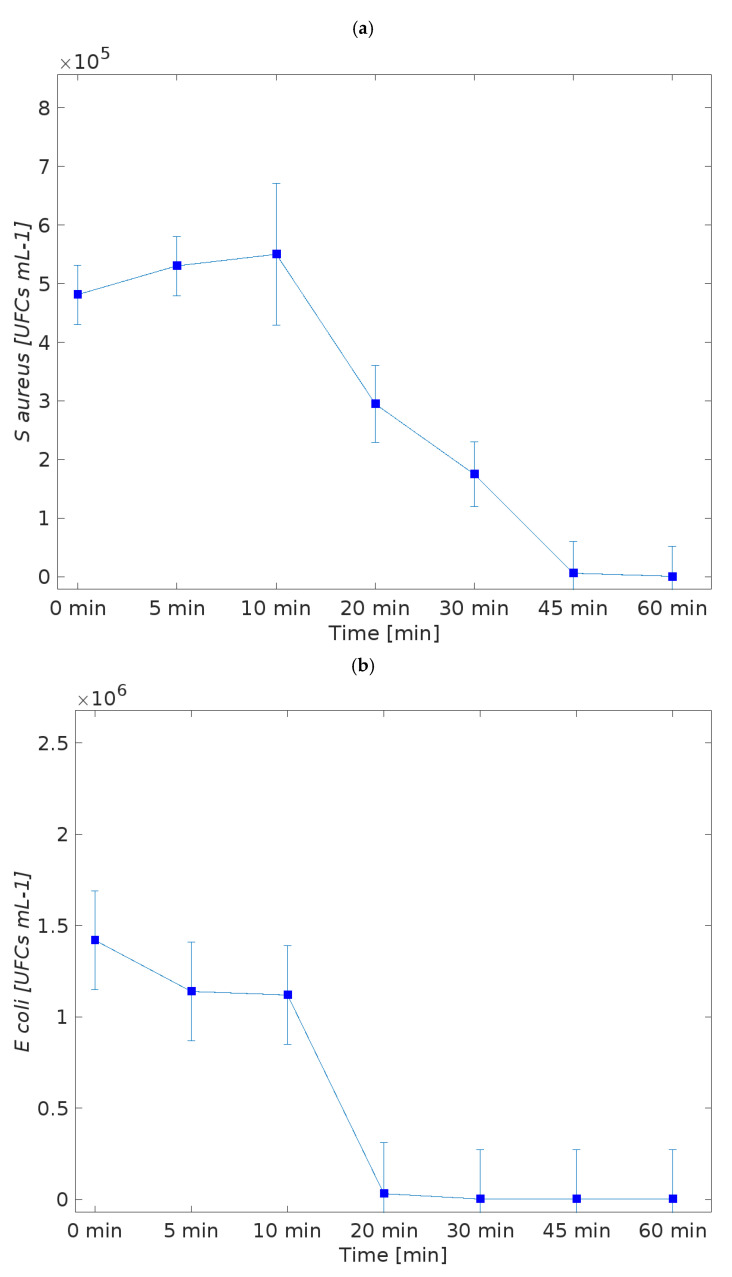
Killing-time curves of (**a**,**b**), the sample functionalized with HKUST-1 against *S. aureus* and *E. coli* bacteria, for up to 60 min of culture. Data derived from two repetitions (means S.D.) according to standards. Positive controls for each bacterium (growth without agent) were also conducted, reaching maximum values of 4.23 × 10^5^ and 2.1 × 10^5^ CFUs mL^−1^ for *S. aureus* bacteria agent-free solution and values of 0.98 × 10^6^ and 1.24 × 10^6^ CFUs mL^−1^ for *E. coli* bacteria agent-free solution, after 1 h (data not shown in graphic).

The antimicrobial activity indicates the presence of copper in the MOF structure, and its intrinsic property as an ion release is the possible mechanism of the antimicrobial action. These results can be observed in the literature, including for textile applications [17,24,36,37]. The kill-time kinetics for the textile sample functionalized with HKUST-1 was determined by the number of remaining viable cells at incubation periods of 0 (before action), 5, 10, 20, 30, 45, and 60 min and the associated log reduction (Figure 3).

The textiles reveal significant inhibitory effects against *E. coli* and *S. aureus*. The results demonstrated that a burst release almost guaranteed the almost complete elimination of the microbial cells over the time tested.

**Figure 4:**
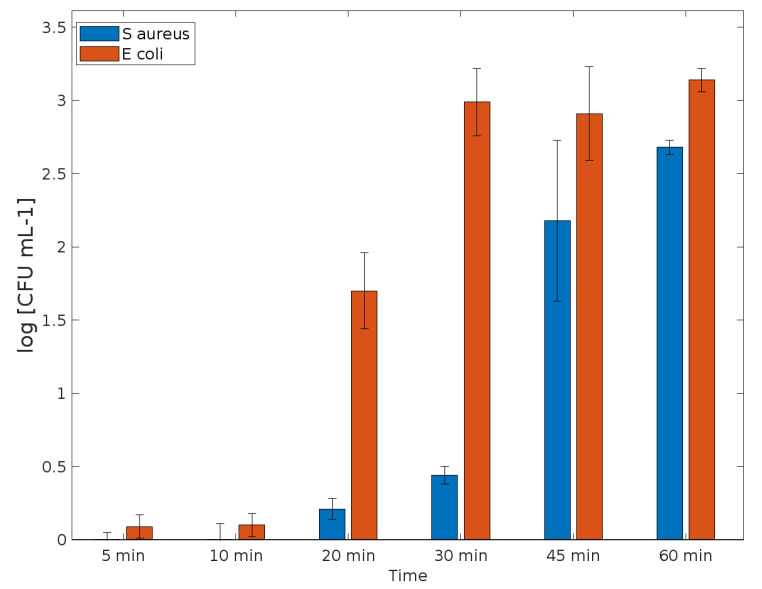
Bacterial log reduction when in contact with the sample functionalized with HKUST-1, after 0, 5, 10, 20, 30, 45 and 60 min. Data derived from two repetitions (means S.D.).

The functionalized samples were capable of presenting an antimicrobial activity in comparison with the control, as expected. Their action begins after 10 min of contact with each bacterium. Data reported an elimination greater than 99.9% for *E. coli* (3.14 log reduction) and bactericidal activity (greater than 3 log_10_ fold decrease in colony forming units). For *S. aureus*, elimination was greater than 99% (2.68 log reduction) after 1 h for the samples treated (Figure 4). Probably, an additional time of the kinect test would present a bactericidal activity as well, once the kinetics demonstrated a greater action after 45 min of contact.

## 4. Conclusions

The search for new materials with antimicrobial activity found in metallic nanomaterials a great ally, due to the high capacity and variability of mechanisms of action of these materials. Therefore, the present work was effective in the production of a textile material with antimicrobial property. From the results obtained, it can be verified that there was a direct synthesis of HKUST-1 in the cotton fabric. SEM images demonstrated a morphology of nanorods that have excellent surface area and crystallinity. It was also found that even after washing, the samples still demonstrated MOF on their surface. These results were reinforced by the analysis of the color of the textile material.

MOF HKUST-1 had its most impactful effect against the bacteria *E. coli* (MIC of 78.125 μg mL^−1^) compared to *S. aureus* (MIC 156.25 μg mL^−1^). On the other hand, functionalized cotton samples demonstrated a similar effect on log reduction, although *E. coli* presented a burst effect after 20 min and *S. aureus* after 45 min.

By mechanical analysis, it was found that there was a considerable drop in the mechanical strength of the textile material, and the temperature and time of the synthesis had a direct and negative relationship with this result. Thus, this work presents one of the ways to carry out the direct synthesis of HKUST-1 onto cotton fabrics. The low mechanical strength of the textile material after finishing tends to reduce its lifespan. Thus, to make the production viable on an industrial scale, the synthesis processes must be re-evaluated.

## Figures and Tables

**Table 1:** Uniaxial force results for all fabric samples and corresponding standard deviation.

Sample	Uniaxial Force (N)	Standard Deviation (N)
Blank	280.80	68.86
1880	74.15	17.84
2480	63.78	7.73
4880	58.84	10.52
18100	45.29	3.98
24100	58.50	9.90
48100	10.97	4.25

**Table 2:** CieLab coordinates results for all samples.

Sample	Cie Lab Coordinates			Blank Deviation			Color Difference
	L	a	b	ΔL	Δa	Δb	ΔE
Blank	89.19 ± 0.26	−1.42 ± 0.16	0.14 ± 0.45	−	−	−	−
1880	87.87 ± 1.43	−9.52 ± 1.59	−0.69 ± 1.20	−1.32	−8.09	−0.82	8.24
2480	85.72 ± 2.62	−11.77 ± 3.81	−3.68 ± 3.90	−3.47	−10.35	−3.81	11.56
4880	87.82 ± 0.83	−10.75 ± 2.13	−1.38 ± 1.14	−1.37	−9.33	−1.52	9.55
18,100	89.55 ± 1.41	−4.88 ± 2.09	2.18 ± 2.01	0.36	−3.46	2.05	4.04
24,100	88.18 ± 2.65	−6.43 ± 1.82	1.00 ± 1.39	−1.01	−5.01	0.87	5.18
48,100	89.06 ± 1.22	−4.72 ± 1.00	2.74 ± 1.12	−0.12	−3.30	2.61	4.21

**Table 3:** Minimum inhibitory concentration (MIC) and Minimum bactericidal concentration (MBC) tested.

	*E. coli*	*S. aureus*
MIC	78.125 μg mL^−1^	156.25 μg mL^−1^
MBC	5 mg mL^−1^	>5 mg mL^−1^

**Table 4:** Antimicrobial activity for samples functionalized with HKUST-1, using quantitative methods.

	N. of Bacteria (CFU mL^−1^) at 0 h (Initial Time)	N. of Bacteria (CFU mL^−1^) at 1 h (Dynamic Contact)	Log_10_ Reduction
*E. coli*	1.42 × 10^6^	1.0 × 10^3^	3.14 ± 0.12
*S. aureus*	5.31 × 10^5^	1.0 × 10^3^	2.68 ± 0.06

Three log reduction means a 99.9% kill of the bacteria.

## Data Availability

Not applicable.
